# Greater fatigability and motor unit discharge variability in human type 2 diabetes

**DOI:** 10.14814/phy2.14503

**Published:** 2020-07-06

**Authors:** Jonathon W. Senefeld, Kevin G. Keenan, Kevin S. Ryan, Sarah E. D'Astice, Francesco Negro, Sandra K. Hunter

**Affiliations:** ^1^ Exercise Science Program Marquette University Milwaukee WI USA; ^2^ Department of Physical Therapy Marquette University Milwaukee WI USA; ^3^ Department of Anesthesiology and Perioperative Medicine Mayo Clinic Rochester MN USA; ^4^ Department of Kinesiology University of Wisconsin Milwaukee WI USA; ^5^ Center for Aging and Translational Research University of Wisconsin Milwaukee WI USA; ^6^ Department of Clinical and Experimental Sciences University of Brescia Brescia Italy

**Keywords:** contractile properties, diabetes mellitus, motor unit behavior, muscle fatigue, steadiness

## Abstract

This study determined the discharge characteristics of motor units from two lower limb muscles before and after fatiguing exercise in people with type 2 diabetes (T2D) with no symptoms of polyneuropathy and activity‐matched controls. Seventeen people with T2D (65.0 ± 5.6 years; 8 women) and 17 controls (63.6 ± 4.5 years; 8 women) performed: (a) intermittent, isometric contractions at 50% maximal voluntary isometric contraction (MVIC) sustained to failure with the ankle dorsiflexors, and (b) a dynamic fatiguing task (30% MVIC load) for 6 min with the knee extensors. Before and after the fatiguing tasks, motor unit characteristics (including coefficient of variation (CV) of interspike intervals (ISI)) were quantified from high‐density electromyography and muscle contractile properties were assessed via electrical stimulation. Fatigability was ~50% greater for people with T2D than controls for the dorsiflexors (time‐to‐failure: 7.3 ± 4.1 vs. 14.3 ± 9.1 min, *p* = .010) and knee extensors (power reduction: 56.7 ± 11.9 vs. 31.5 ± 25.5%, *p* < .001). The CV of ISI was greater for the T2D than control group for the tibialis anterior (23.1 ± 11.0 vs. 21.3 ± 10.7%, *p* < .001) and vastus lateralis (27.8 ± 20.2 vs. 24.5 ± 16.1%, *p* = .011), but these differences did not change after the fatiguing exercises. People with T2D had greater reductions in the electrically evoked twitch amplitude of the dorsiflexors (8.5 ± 5.1 vs. 4.0 ± 3.4%·min^‐1^, *p* = .013) and knee extensors (49.1 ± 10.0 vs. 31.8 ± 15.9%, *p* = .004) than controls. Although motor unit activity was more variable in people with T2D than controls, the greater fatigability of the T2D group for lower limb muscles was due to mechanisms involving disruption of contractile function of the exercising muscles rather than motor unit behavior.

## Graphical abstract

We show that men and women with type 2 diabetes (T2D) and no signs of polyneuropathy exhibit greater fatigability, reduced force steadiness, and greater variability in single motor unit activity of the ankle dorsiflexors and knee extensor muscle than controls matched for age, activity, and body mass index. The T2D‐associated fatigability was not correlated with greater motor unit discharge variability but associated with reductions in contractile properties of both lower limb muscle groups.

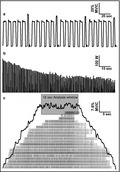

**Abbreviations**
ANOVA: analysis of variance
CV: coefficient of variation
EMG: electromyography
HbA_1c_: glycated hemoglobin
ISI: Interspike Interval
min: minutes
MVCC: maximal voluntary concentric contraction
MVIC: maximal voluntary isometric contraction
T2D: type 2 diabetes mellitus

## 1 INTRODUCTION

Type 2 diabetes mellitus (T2D) is characterized by chronically elevated blood glucose and is one of the most costly chronic diseases in the United States (Trogdon et al., 2015). Although physical activity is a cornerstone of management of T2D (Sigal, Kenny, Wasserman, and Castaneda‐Sceppa (2004)), there are numerous musculoskeletal complications associated with T2D that create a significant barrier to complete regular exercise (Smith, Burnet, & McNeil, 2003). These musculoskeletal complications include reduced maximal muscle strength and power (Ijzerman et al., 2011) and greater fatigability of limb muscles (activity‐induced reductions in strength or power (Gandevia, 2001; Hunter, 2018)) during exercise compared with healthy controls (Allen, Kimpinski, Doherty, & Rice, 2015; Almeida, Riddell, & Cafarelli, 2008; Bazzucchi et al., 2015; Halvatsiotis, Short, Bigelow, & Nair, 2002; Ijzerman et al., 2012; Petrofsky et al., 2005; Senefeld, Limberg, Lukaszewicz, & Hunter, 2019; Senefeld, Magill, Harkins, Harmer, & Hunter, 2018). These impairments in skeletal muscle function are further exacerbated by diabetic polyneuropathy (DPN) especially within motor neurons of the lower limb in people with T2D (Ijzerman et al., 2011).

Diabetic polyneuropathy is a peripheral nerve dysfunction resulting in axonal loss or demyelination that begins and remains more pronounced in distal portions of the lower limb, and then successively affects proximal regions (Allen, Doherty, Rice, & Kimpinski, 2016; Said, 2007; Senefeld & Hunter, 2016). Thus, comparison of an upper and lower limb muscle group or proximal and distal muscle group often serve as a proxy to estimate the effects of DPN (Said, (2007)). There are a host of functional impairments of motor neurons in people with T2D and DPN that cumulatively result in a progressive reduction in skeletal muscle strength and power (Allen, Choi, Kimpinski, Doherty, & Rice, 2013; Allen, Kimpinski, et al., 2015; Allen, Stashuk, et al., 2015; Almeida et al., 2008; Andersen, Stalberg, Gjerstad, & Jakobsen, 1998; Bazzucchi et al., 2015; Butugan et al., 2014; Chang & Chuang, 1996; Miglietta, 1973). Motor neuron dysfunction and subsequent motor impairments due to DPN are believed to be the primary mechanism for greater fatigability in people with T2D and DPN compared with controls (Allen et al., 2013, 2016; Allen, Kimpinski, Doherty, & Rice, 2014; Allen, Kimpinski, et al., 2015; Allen, Major, Kimpinski, Doherty, & Rice, 2014; Allen, Stashuk, et al., 2015). Thus, it is not surprising that people with T2D and DPN exhibit lower and more variable discharge frequencies of motor units than controls during motor tasks (Watanabe et al., 2013; Watanabe, Miyamoto, Tanaka, Fukuda, & Moritani, 2012). Whether motor unit behavior differs in people with T2D who do not have clinical signs of DNP is not known.

Clinical detection of DPN in people with T2D typically requires overt sensory loss in the feet and/or hands possibly subsequent to alterations or even subclinical changes in the motor neuron (Stino & Smith, 2017). Thus, it is suggested that the motor neurons changes associated with DPN may contribute to loss of muscle strength and greater fatigability of people with T2D *without* clinically detected DPN (Allen et al., 2016); however, there are limited empirical findings to support this suggestion. It is not known whether motor unit behavior is altered in people with T2D without DPN, or if any change in motor unit behavior affects motor control tasks such as torque steadiness, as well as muscle strength and fatigability of limb muscles in people with T2D *without* DPN.

The purpose of the study was to compare fatigability, torque steadiness, and motor unit discharge properties of two lower limb muscle groups between people with T2D and a control group. Our *hypotheses* were that compared to a healthy control group matched for physical activity, body mass index, age, and gender, people with T2D and without clinically detected DPN would demonstrate: (a) greater fatigability and lower torque steadiness of both lower limb muscle groups, (b) greater variability of motor unit discharge, and (c) the greater variability of motor unit discharge would be exacerbated after fatiguing contractions. We chose two different fatiguing tasks (isometric and dynamic contraction tasks) because fatigability of limb muscles and the contributing mechanisms are dependent on the details of the task in many populations (Hunter, 2018; Senefeld, Pereira, Elliott, Yoon, & Hunter, 2018b). Thus, while we kept the contraction task by which motor unit behavior was determined (isometric ramp tasks) similar before and after the two fatiguing contractions, we varied the fatiguing contraction type. To understand single motor unit behavior we used high‐resolution electromyography (EMG), to quantify motor unit discharge characteristics of both a proximal (knee extensors) and distal (ankle dorsiflexors) lower limb muscle group. Comparison of the effects on a proximal and distal muscle group served as a proxy to estimate the effects of changes in motor neuron function due to undetected DPN, which should be exacerbated in the more distal muscle group (Said, 2007).

## 2 MATERIALS AND METHODS

Seventeen people with T2D (65.0 ± 5.6 years, 8 females) and 17 controls (63.6 ± 4.5 years, 8 females) matched for age, gender, body fat, and physical activity participated in the study. Participants provided written informed consent and the protocol was approved by the Marquette University Institutional Review Board (HR‐2402) in accordance with the Declaration of Helsinki for human experimentation. T2D was physician diagnosed and was known prior to study enrollment and participation. As we have used for previous studies (Senefeld, Harmer, & Hunter, 2019; Senefeld, Limberg, et al., 2019; Senefeld, Magill, et al., 2018), exclusion criteria included unstable glycemic control (HbA_1c_ > 10%), hemoglobinopathies, iron deficiency, hemolytic anemia, diabetic neuropathy (assessed via clinical diagnosis, monofilament and tuning fork sensation tests, and sensory questionnaires), peripheral edema, severe obesity (body mass index >45 kg m^‐2^), untreated hypothyroidism, epilepsy, medications that affect cortical excitability, current smoking, possibility of pregnancy, and any neurological, cardiovascular, or musculoskeletal disease that precluded exercise testing.

Four people were excluded from the dorsiflexor muscles experimental session. One man with T2D had a prosthetic hallux due to congenital hammer toe which resulted in labored and highly variable dorsiflexion torque. One woman in the control group was unable to perform a dorsiflexion task without significant activation of the hip flexor muscles. Two males in the control group had cramping of the plantar flexor muscles during baseline testing of maximal voluntary isometric contractions (MVIC) and submaximal tracing tasks and unable to perform the tasks. Thus, 16 participants with T2D (*n* = 7 females) and 14 controls (*n* = 7 females) completed the dorsiflexor muscles experimental session and are included in the analysis.

Participants completed three sessions of testing that included a screening and familiarization session to determine eligibility and become familiar with experimental procedures for the study followed by two experimental sessions. In brief, the experimental sessions included measurements of maximal strength, torque steadiness, contractile function, and motor unit characteristics of either the ankle dorsiflexor muscles or knee extensors muscles before and after performance of a fatiguing contraction task of the same muscle group. The fatiguing task for the ankle dorsiflexors was an intermittent isometric contraction performed at 50% of MVIC until failure (as defined below) and for the knee extensor muscles was 6 min of repeated fast‐velocity knee extensions with a load equivalent to 30% of MVIC.

### 2.1 Screening and familiarization session

The following tests were performed using standard methods, as we have previously reported (Senefeld, Harmer, et al., 2019; Senefeld, Limberg, et al., 2019; Senefeld, Magill, et al., 2018), including: (a) diabetic polyneuropathy screen, (b) HbA_1c_ point‐of‐care assay, (c) anthropometrics via DEXA scan, and (d) estimation of peak aerobic capacity with graded bicycle ergometer exercise test. These data are presented in Table 1. Leg dominance was determined by preferred kicking foot (Kelley, He, Menshikova, & Ritov, 2002; Laughlin, 2016); all participants reported right leg dominance. Participants then practiced muscle contractions that were central to each protocol, including brief MVICs with both muscle groups, intermittent isometric contractions at 50% MVIC with the dorsiflexors and maximal voluntary (fast‐velocity) concentric (shortening) contractions (MVCCs) with the knee extensor muscles.

**TABLE 1:** Physical characteristics of people with T2D and controls

Variable	Units	Type 2 Diabetes	Controls	*p*‐value
		*n = 17*	*n = 17*	
Age	years	65.0 ± 5.6	63.6 ± 4.5	.352
Body Fat	%	37.8 ± 7.8	34.4 ± 7.0	.143†
estimated VO_2_	mLO_2_·kg^−1^·min^−1^	23.8 ± 4.9	29.0 ± 9.4	.052
Physical Activity	steps·day^−1^	8,780 ± 5,072	8,028 ± 3,217	.867
HbA_1c_	%	7.63 ± 1.21	5.85 ± 0.26	<.001*
Thigh Muscle Mass	kg	7.39 ± 1.28	7.12 ± 1.04	.139†
Shank Muscle Mass	kg	1.72 ± 0.84	1.48 ± 0.53	.167†

Note

Body fat was measured via dual X‐ray absorptiometry, estimated VO_2_ was measured via submaximal, graded bicycle test, physical activity was measured via triaxial accelerometry, and HbA_1c_ was measured via point‐of‐care instrument assay.

Abbreviations: HbA_1c_, glycated hemoglobin; VO_2_, maximal aerobic capacity.

* Denotes group‐related difference between controls and T2D, with *p*‐value listed.

^†^ Denotes gender‐related difference, *p* < .05.

### 2.2 Experimental sessions

The two experimental sessions involved performance of one of two fatiguing tasks: (a) an intermittent isometric task with the ankle dorsiflexor muscles performed at 50% MVIC (6‐s contraction, 4‐s rest) until task failure (as defined below) or (b) a dynamic fatiguing task with the knee extensor muscles involving 120 MVCCs (1 every 3 s) performed over 6 min with a load equivalent to 30% MVIC torque. The order of the sessions was randomized. Fatigability of the ankle dorsiflexors was considered the time to failure of the 50% MVIC task. Fatigability of the knee extensors was considered the percent reduction in knee extensor power from average of the first five contractions (1‐5) relative to the average of the last five contractions (116‐120).

Before and after each of the fatiguing tasks, the following assessments were performed on the agonist muscle group (ankle dorsiflexor or knee extensor muscles): (a) MVIC trials to determine maximal strength; (b) the interpolated twitch technique using electrical stimulation of the motor nerve during repeated MVICs to determine voluntary activation; (c) electrically evoked contractions of the motor nerve to measure muscle contractile properties; and (d) single motor unit recording using high‐density surface electrodes during isometric contractions performed at 10% and 40% of MVIC to determine torque steadiness and motor unit discharge characteristics (discharge rate frequency and variability).

### 2.3 Experimental set Up

Each participant performed all contractions with the dominant leg while seated in a Biodex System 4 dynamometer (Biodex Medical). For the knee extensor muscles experimental session, participants were seated with 90° of hip and knee flexion with tightly secured, padded straps across each shoulder and the waist to limit ancillary movement. The leg was positioned such that the axis of rotation of the knee joint was aligned with the axis of rotation of the dynamometer, and the shank of the leg was secured to the lever arm of the dynamometer with a Velcro strap ~4 cm proximal to the lateral malleolus.

For the dorsiflexor muscles experimental session, participants were positioned with 90 degrees of ankle flexion and the axis of rotation of the ankle joint was aligned with the axis of rotation of the dynamometer. Straps mounted to the foot attachment were tightly secured across the dorsum of the foot. Participants were seated in a reclined posture (30° relative to upright) with 90° of hip and knee flexion and a padded support strapped to the thigh to limit hip flexion because hip flexion torque has the potential to be recorded as dorsiflexion torque. To confirm that hip flexor activation was minimal, a custom‐made, weighted heel pad was placed under the calcaneus of the participants that provided auditory feedback if the calcaneus was raised off the footplate. All dorsiflexion contractions were performed at 30° of plantar flexion.

#### 2.3.1 Muscle strength and contractile function

At the beginning of each session, maximal strength of either the ankle dorsiflexor muscles or the knee extensors muscles was determined with three brief MVICs (~3 s) without stimulation to the motor nerve. Additional MVICs were performed if the participant did not achieve two MVICs within 5%. At least 1‐min rest was provided between each MVIC trial.

After the initial three MVICs, supramaximal electrical stimulation was used to elicit a twitch contraction. Electrically evoked twitch contractions were elicited during each MVIC (superimposed) and immediately after each MVIC (<1.5 s), while the muscle was in a potentiated state (to determine contractile function and voluntary activation, see ‘data analysis’ section) and voluntary torque had returned to zero. At least 2‐min rest was provided between each MVIC with the interpolated electrical stimulation.

#### 2.3.2 Electrical stimulation

##### Dorsiflexors

To assess voluntary activation and the potentiated twitch amplitude, the dorsiflexor muscles were stimulated with a double pulse (100 Hz, 200 µs duration, 400 V, 65–240 mA) via constant‐current stimulator (DS7AH; Digitimer, Ltd.). The cathode electrode (Ambu Neuroline electrodes, Denmark; 1.5 cm diameter) was placed ~2 cm distal and ~2 cm medial to the fibular head and the anode was placed ~2 cm distal to the fibular head. The intensity of the nerve stimulation was determined by increasing the current of the stimulator until the twitch amplitude plateaued (<5% increase between twitch contraction amplitude) and there were no palpable or visible signs of antagonist (plantar flexor) activation.

##### Knee extensors

The femoral nerve innervating the knee extensor muscles was stimulated using supramaximal intensities (180–500 mA) with a single pulse (200 µs duration, 400 V) to elicit a potentiated twitch. The cathode electrode (Ambu Neuroline electrodes) was placed over the femoral nerve within the femoral triangle and the anode electrode was placed over the greater trochanter of the femur. The stimulator intensity was determined by increasing the current until the potentiated twitch amplitude plateaued (<5% increase between twitch contraction amplitude), then the stimulation intensity was increased further by 20% to ensure a maximal excitation of the femoral nerve.

#### 2.3.3 Isometric fatiguing task session with dorsiflexor muscles

**FIGURE 1:**
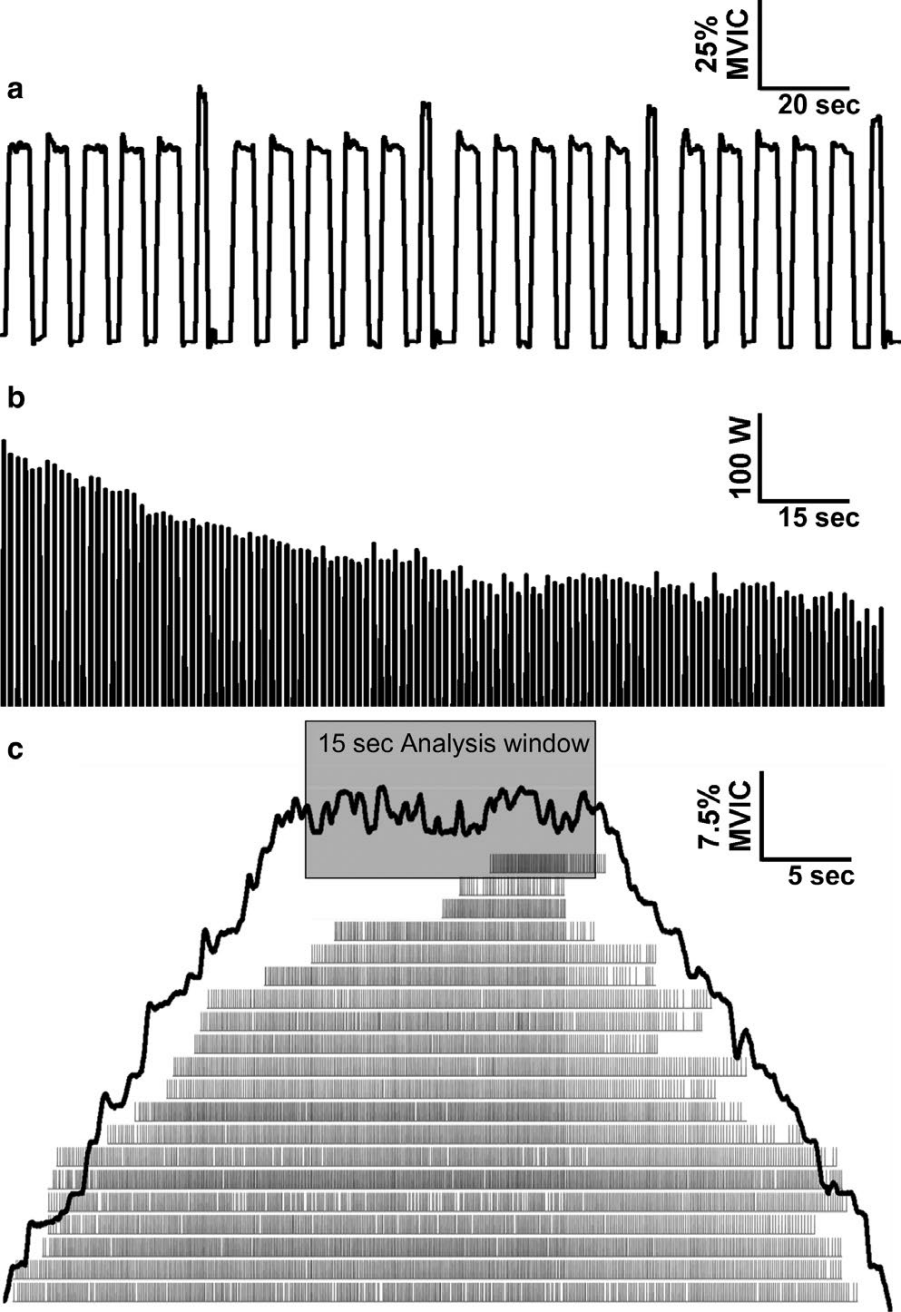
Representative muscle performance data from a 62‐year‐old man with type 2 diabetes. (a) Dorsiflexor torque during an intermittent, isometric fatiguing task. (b) Knee extensor power during the 6‐min dynamic fatiguing task. (c) Isometric knee extension torque (black line) and motor unit discharge times (vertical grey bars) of 20 verified motor units during a submaximal (40% MVIC) tracing task. Abbreviations: MVIC, maximal voluntary isometric contraction; W, Watts

The intermittent isometric fatiguing protocol involved 6‐s contractions with the ankle dorsiflexor muscles at 50% MVIC, each contraction followed by 4‐s rest intervals. A 6‐s MVIC was performed every minute (in lieu of 50% MVIC), followed by 4 s of rest. Each participant was verbally encouraged throughout the protocol. Task failure was identified as the time when the torque dropped below 50% MVIC for more than 3 s of the 6‐s contraction. After task failure was attained, an MVIC was performed as the next contraction and then the fatiguing task was terminated. See Figure 1A.

#### 2.3.4 Dynamic fatiguing task session with knee extensor muscles

After the baseline MVICs, participants performed 10 MVCCs with a load equivalent to 30% MVIC with the knee extensor muscles. MVCCs were performed through an 85º range of motion (90º of knee flexion to 5º of knee extension). After 3 min of rest, the dynamic fatiguing protocol was initiated. The fatiguing protocol involved 120 MVCCs of the knee extensor muscles through an 85º range of motion with one MVCC every 3 s (6 min total). Individuals actively performed the concentric phase for knee extension and the dynamometer passively returned the limb to the initial position at 90º of knee flexion. Participants were strongly encouraged to maintain maximal effort throughout the dynamic fatiguing task. See Figure 1B.

#### 2.3.5 Submaximal contractions

Torque steadiness and motor unit discharge characteristics were assessed during submaximal isometric contractions at 10% and 40% baseline MVIC performed before and after the fatiguing tasks. The order of contraction intensity (10% and 40% MVIC) was randomized at each time point, and these two intensity contractions were selected to examine low recruitment threshold (10% MVIC) and high recruitment threshold (40% MVIC) motor units. For both muscle groups, these submaximal contractions were initiated within 10‐s after completion of the fatiguing task, immediately after an MVIC. Participants traced trapezoidal torque trajectories presented on a computer screen. The trapezoidal torque trace consisted of a 15‐s constant positive slope, 15‐s plateau, and 15‐s constant negative slope with the plateau at 10% or 40% baseline MVIC. Trapezoidal torque traces at each torque intensity were performed twice before and after the fatiguing task (isometric or dynamic), with 10‐s rest between each contraction.

Motor unit recruitment and derecruitment thresholds (percentage MVIC) were determined during the ‘ramp phase’ of the trapezoidal torque trace contractions with a 40% MVIC plateau during the 15‐s positive slope and 15‐s negative slope, respectively. Motor unit discharge properties were determined during the constant‐torque segment of each contraction. See Figure 1C.

#### 2.3.6 Multichannel electromyography (EMG)

##### Multichannel EMG recordings

During the submaximal contractions, multichannel surface EMG signals were recorded from one agonist muscle (vastus lateralis or tibialis anterior) with a semidisposable adhesive grid of 64 electrodes (ELSCH064NM2; OT Bioelettronica, Torino, Italy). Each electrode grid was comprised of 13 rows and 5 columns of electrodes (1 mm diameter, 8 mm interelectrode distance) with the upper left corner of the grid having no electrode. A double‐sided adhesive matrix foam (KITAD064NM2; OT Biolettronica) was adhered to the EMG matrix, individual apertures overlying each electrode were filled with conductive paste, the matrix foam was adhered to the participants’ skin, connected to a 64‐channel single adapter (AD1x64), and secured with self‐adherent wrap. The electrode grid was positioned over the muscle belly and in line with the muscle fiber orientation in the midpoint of the longitudinal axis for the vastus lateralis (Watanabe et al., 2013) and ~3 cm distal to the tibial tuberosity for the tibialis anterior. Two reference electrodes (WS2; OT Bioelettronica) were positioned on the malleolus of the nonexercising leg. EMG signals were digitized at 2048 Hz with a 12‐bit analog‐to‐digital converter (EMG‐USB 2, Bioelettronica, Torino, Italy, 3dB, bandwidth 10‐500 Hz). EMG signals were amplified by a factor of 1,000. Analog recordings from the dynamometer corresponding to torque were digitized and recorded (512 Hz; EMG‐USB 2) and referenced for the EMG analyses.

#### 2.3.7 Data analysis: Multichannel EMG signals

Monopolar EMG and torque signals were exported and analyzed offline using custom analysis algorithms (MATLAB version R2016b; The MathWorks). Each of the 64 channels of EMG was visually examined to remove channels saturated with excessive electrical noise or containing no EMG signal. The remaining channels were decomposed using a validated multichannel convolutive blind source separation algorithm (Martinez‐Valdes et al., 2017; Negro, Muceli, Castronovo, Holobar, & Farina, 2016a) to discriminate individual motor unit action potentials. Each decomposed signal was examined to exclude transient waveforms based on criterion developed from work on single motor units (Barry, Pascoe, Jesunathadas, & Enoka, 2007; Moritz, Barry, Pascoe, & Enoka, 2005; Pascoe, Holmes, Stuart, & Enoka, 2014) and commonly used in multichannel EMG signal analysis (Mani et al., 2018). Decomposed signals were excluded for the following reasons: insufficient motor unit discharges (*n* < 50), nonphysiological range of interspike interval (ISI) (ISI < 25 ms or > 400 ms), or nonphysiological range of coefficient of variation (CV) of ISI (ISI < 10% or > 50%). For the tibialis anterior, 304 (6.9%) waveforms were excluded from an original 4,406 decomposed signals and 4,102 (93.1%) motor units were used for analysis. For the vastus lateralis, 200 (19.7%) waveforms were excluded from an original 1,014 decomposed signals and 814 (80.3%) motor units were used for analysis. The number of identified motor units was much less for the vastus lateralis compared to the tibialis anterior as commonly observed (Holobar, Farina, Gazzoni, Merletti, & Zazula, 2009) likely due to greater thickness of subcutaneous fat which diminishes the signal in the upper leg compared to lower leg. It is noteworthy that for vastus lateralis recordings among females, 87 waveforms were decomposed, and all 87 waveforms were excluded. Thus, motor unit data for the vastus lateralis were analyzed and reported using males only.

#### 2.3.8 Data analysis: Torque, velocity, and position

Analog mechanical recordings from the dynamometer corresponding to torque for the dorsiflexor and knee extensor muscles, and velocity and position for the knee extensor muscle session were digitized and recorded (512 Hz) with a Power 1,401 analog‐to‐digital converter and Spike2 software (Cambridge Electronics Design).

For both muscle groups, maximal torque during each MVIC was quantified as the average value over a 0.1 s interval prior to the onset of the electrical stimulation. Torque steadiness was quantified over the middle 7.5 s interval of the 15 s plateau in the trapezoidal torque trajectory as the amplitude of the normalized torque fluctuations using the CV of torque: [CV = (standard deviation of torque) · (mean of torque)^‐1^·100%]. The amplitude of the potentiated twitch from the electrically evoked contractions was measured as the maximal torque response in a 250‐ms window after the stimulation pulse. Voluntary activation was calculated as the ratio of the amplitude of the superimposed twitch (SIT) during the MVIC to the amplitude of the potentiated twitch elicited at rest following the MVIC with the following equation: voluntary activation = (1 – SIT · Potentiated Twitch^‐1^) · 100% (Gandevia, 2001).

For the knee extensor muscles, the maximal angular power during dynamic contractions was quantified as the maximal value during the concentric phase of the contraction (MVCC power) and is presented as the average from five consecutive contractions at Baseline (contractions 1–5). Fatigability of the knee extensor muscles during the 120 repeated MVCCs was the percentage change in the MVCC power from Baseline (contractions 1–5) to Task end (contractions 116–120). Fatigability for the ankle dorsiflexor muscles was considered the time‐to‐task failure of the intermittent isometric fatiguing task.

The relative change in variables (Δ) from before to after the fatiguing task was calculated as: Δ = [1 – (Task End value · Baseline value^‐1^)] · 100%; including MVIC torque, potentiated twitch amplitude, torque steadiness, and motor unit characteristics (de/recruitment thresholds, mean ISI, and CV for ISI). Because voluntary activation is characteristically a relative value, the relative change (Δ) was calculated as: Δ = Task End value – Baseline value.

For the isometric fatiguing task with the dorsiflexor muscles, the time‐to‐task failure was a dependent variable, and therefore, to compare the change in performance (MVIC torque, voluntary activation, and potentiated twitch amplitude), the average relative rate of change (%·min^‐1^) was calculated as: (relative change, Δ) · (time to failure)^‐1^.

### 2.4 Statistical analysis

Individual univariate analyses of variance (ANOVAs) were used to compare participant characteristics, baseline measures of muscle function, and time‐to‐task failure of the ankle dorsiflexor muscles with group (T2D and control) and gender (males and females) as between‐subject factors. Repeated‐measures ANOVAs were performed on torque variability (CV of torque) during submaximal contractions (10% and 40% MVIC), the measures of fatigability (MVCC power for the knee extensor muscles, and MVIC torque for both muscle groups) and the accompanying mechanistic measurements (electrically evoked twitch amplitude and voluntary activation) with time and contraction intensity as within‐subject factors and group and sex as between‐subject factors. Prior to statistical comparisons, normality of distributions and homogeneity of variance of the data were assessed using the Kolmogorov–Smirnov test and Levene's statistic, respectively.

The number of motor unit that were able to be analyzed was larger in number for the tibialis anterior (*n* = 4,102) than the vastus lateralis (*n* = 814) and differed between participants (*n* = 1‐ 40) at each time point (before and after fatiguing task). To test for differences in motor unit characteristics (number, de/recruitment threshold, mean ISI, and CV for ISI), a nested ANOVA was used with group (T2D and control), gender (males and females), time (pre‐ and post‐fatigue), and intensity (10% and 40% MVIC) as fixed factors.

Separate Pearson correlation coefficients (*r*) were used to determine associations between the primary measures of fatigability (time to failure or reductions in MVCC power) and participant characteristics, baseline muscle characteristics, motor unit characteristics, and the potential mechanisms contributing to fatigability. The variables that were significantly correlated were entered into a stepwise, linear, multiple‐regression analysis to identify those characteristics most closely associated with the variance in measures of fatigability. For each multiple regression model, only one variable was selected in the final regression model, thus, curve estimation model regression analyses were used to identify the optimal prediction model of fatigability. All significance levels were set a priori at *p* < .05 and all statistical analyses were performed using IBM Statistical Package for Social Sciences (SPSS, version 25, Armonk, NY). Data are presented as the mean ± standard deviation (SD) in the text and tables.

## 3 Results

### 3.1 Baseline

Participants were group‐matched according to age, body, fat, and physical activity (see Table 1). The people with T2D and controls did not differ in MVIC strength, voluntary activation, and electrically evoked twitch amplitude for either the ankle dorsiflexor or knee extensor muscles as reported in Table 2. However, people with T2D had greater MVCC power of the knee extensor muscles than controls.

**TABLE 2:** Muscle function and fatigability of people with T2D and controls

Variable	Units	Dorsiflexors			Knee Extensors		
		T2D	Control	*p*‐value	T2D	Control	*p*‐value
		*n = *16	*n = *14		*n = *17	*n = *17	
Time to task failure	min	7.3 ± 4.1	14.3 ± 9.1	.01	—	—	
MVCC power							
Baseline	W	—	—		299.0 ± 132.9	232.5 ± 66.4	.005* ^,^ †
Task End	W	—	—		128.2 ± 57.8	157.1 ± 76.7	.18†
Δ	%	—	—		55.6 ± 12.4	31.5 ± 25.5	.002*
MVIC torque							
Baseline	Nm	31.1 ± 9.7	34.7 ± 7.4	.217†	165.3 ± 71.1	158.8 ± 67.6	.442†
Task End	Nm	20.8 ± 6.1	23.8 ± 6.4	.172†	85.0 ± 35.8	94.4 ± 35.7	.309†
Δ	%	32.7 ± 9.2	31.4 ± 9.0	.714	46.9 ± 11.3	35.8 ± 9.8	.013*
***Twitch amplitude***							
Baseline	Nm	6.10 ± 3.03	7.31 ± 3.06	.158†	57.1 ± 24.9	52.1 ± 18.1	.701†
Task End	Nm	3.21 ± 1.39	4.50 ± 1.92	.549†	28.1 ± 11.1	35.6 ± 15.6	.008* ^,^ †
Δ	%	42.8 ± 19.9	37.3 ± 15.9	.371	49.1 ± 10.0	31.8 ± 15.9	.004*
Voluntary activation							
Baseline	%	97.9 ± 1.7	98.5 ± 1.0	.212	95.9 ± 4.2	95.0 ± 5.8	.299
Task End	%	95.2 ± 3.8	96.0 ± 3.9	.575	93.6 ± 6.8	95.3 ± 4.2	.629
Δ	%	2.8 ± 4.7	2.5 ± 4.0	.859	—	—	

Note

Metrics of fatigability are displayed, including baseline measures (Baseline), measures immediately after the fatiguing task (Task End), and the relative reduction during or after the fatiguing task (Δ). Values are displayed as mean ± SD. ‐ ‐ denotes data that were not measured (time to task failure for knee extensors; MVCC power for dorsiflexors) or metrics that did not change after the fatiguing task (voluntary activation, knee extensors).

Abbreviations: MVCC, maximal voluntary concentric contractions, MVIC, maximal voluntary isometric contractions; T2D, type 2 diabetes.

* Denotes group‐related difference between controls and T2D, *p* < .05.

^†^ Denotes gender‐related difference, *p* < .05.

### 3.2 Fatigability 

#### 3.2.1 Dorsiflexor muscles 

**FIGURE 2:**
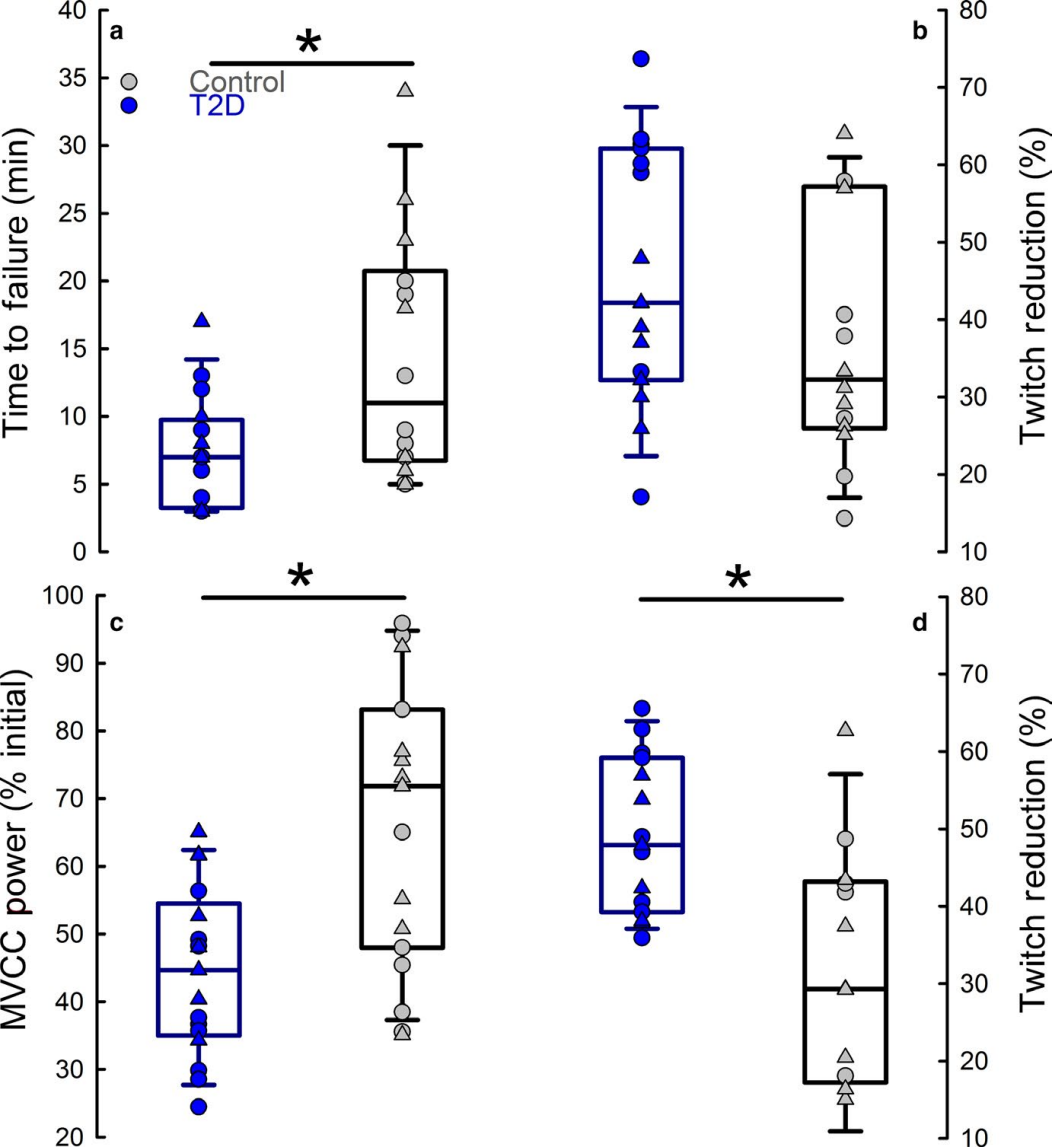
Fatigability and Reductions in Electrically Evoked Twitch Amplitude for the Dorsiflexor and Knee Extensor Muscles. Fatigability was ~2× greater for people with T2D (blue symbols) compared to controls (grey symbols)—time‐to‐task failure for the static dorsiflexor fatiguing task (a) and the MVCC power at the end of the dynamic knee extensor fatiguing task (c). The reduction in electrically evoked twitch amplitude after the fatiguing task was not different (*p* = .371) for the dorsiflexor task (b) but was greater for people with T2D compared to controls after the knee extensor task (d). * denotes group difference between people with T2D and controls, *p* < .05. Men are denoted in circles and women are denoted in triangles

People with T2D had a shorter time‐to‐task failure (i.e., greater fatigability, 51% difference) compared with the control group (Table 2). The reduction in MVIC torque at task failure was not different between the two groups. Additionally, there were no group‐related differences in the reduction in electrically evoked twitch amplitude or voluntary activation. However, the average relative rate of decline in MVIC torque (6.2 ± 4.1 vs. 3.5 ± 2.7%·min^‐1^, *p* = .049) and electrically evoked twitch amplitude (8.5 ± 5.1 vs. 4.0 ± 3.4%·min^‐1^, *p* = .013) were greater for people with T2D than the controls. In contrast, the average rate of decline was not different between groups for voluntary activation (0.32 ± 0.70 vs. 0.32 ± 0.73%·min^‐1^, *p* = .994). See Table 2 and Figure 2A,B.

#### 3.2.2 Knee Extensor muscles

People with T2D had greater reductions in knee extensor power during the dynamic fatiguing task than controls (55.6% vs. 31.5%, respectively) and larger declines in MVIC torque (46.9% vs. 35.8%, respectively) after the dynamic exercise task (i.e. greater fatigability). Additionally, people with T2D had greater reductions in electrically evoked twitch amplitude compared with controls (49.1% vs. 31.8%, respectively). However, both groups demonstrated no reduction in voluntary activation (time effect, *p* = .189; time × group, *p* = .495). See Table 2 and Figure 2C,D.

### 3.3 Submaximal ramp contractions: Torque variability and motor unit characteristics

#### 3.3.1 Dorsiflexor muscles

##### Torque variability

The CV of dorsiflexor torque was greater during the 10% MVIC task than the 40% MVIC task (*p* < .001, main effect of contraction intensity). People with T2D had a greater CV of torque compared with the controls for both contraction intensities (10% and 40% MVIC, Table 3). The CV of torque during the 10% MVIC task did not differ after the fatiguing task (*p* = .897); however, for the 40% MVIC task, the CV of torque decreased after the fatiguing task (*p* = .015).

**TABLE 3:** Torque steadiness of the dorsiflexor (DF) and knee extensor (KE) muscles, and motor unit numbers and discharge characteristics for the tibialis anterior and vastus lateralis at two target torques (10% and 40% MVIC) before and after the fatiguing tasks

Variable	Units	T2D		Control		*p*‐value
		Before	After	Before	After	
Tibialis Anterior						
DF CV of Torque						
10% Task	%	12.39 ± 11.63	12.56 ± 9.25	9.73 ± 4.33	9.16 ± 4.31	.027^†^ ^,^ ^‡^
40% Task	%	6.21 ± 4.03	4.37 ± 2.58	4.61 ± 2.63	3.78 ± 1.54	.024* ^,^ ^†^
Motor Units						
10% Task	*N*	478	452	452	478	
40% Task	*N*	597	544	558	543	
Mean ISI						
10% Task	ms	100.0 ± 20.8	96.6 ± 19.0	98.4 ± 18.7	95.4 ± 16.2	.099* ^,^ ^‡^
40% Task	ms	69.8 ± 12.7	67.9 ± 13.0	70.8 ± 13.9	68.3 ± 14.3	.583*
CV for ISI						
10% Task	%	18.0 ± 9.5	21.8 ± 12.3	17.9 ± 10.4	19.5 ± 12.3	.037* ^,^ ^†^ ^,^ ^‡^
40% Task	%	24.9 ± 10.2	26.6 ± 10.2	23.1 ± 9.0	23.9 ± 10.1	<.001* ^,^ ^†^
Vastus Lateralis						
KE CV of Torque						
10% Task	%	3.45 ± 1.18	3.96 ± 1.70	3.24 ± 1.23	3.38 ± 1.14	.043^†^
40% Task	%	3.00 ± 1.07	3.72 ± 1.98	2.37 ± 0.81	2.30 ± 0.79	<.001^†^
Motor Units						
10% Task	*n*	113	85	99	90	
40% Task	*n*	140	78	107	102	
Mean ISI						
10% Task	ms	114.6 ± 21.4	112.1 ± 26.9	116.2 ± 29.3	112.2 ± 27.4	.723* ^,^ ^‡^
40% Task	ms	90.7 ± 17.9	79.0 ± 17.7	101.3 ± 23.2	91.7 ± 23.2	<.001* ^,^ ^†^
CV for ISI						
10% Task	%	23.3 ± 17.6	26.9 ± 22.1	22.0 ± 17.3	24.1 ± 18.8	<.001* ^,^ ^‡^
40% Task	%	29.7 ± 20.0	31.2 ± 20.0	25.8 ± 14.6	27.0 ± 12.0	<.001* ^,^ ^†^

Note

Values are displayed as mean ± SD. Target torques were isometric ramp contractions relative to maximal voluntary isometric contraction (MVIC) torque.

Abbreviations: CV, coefficient of variation; ISI, interspike interval; T2D, type 2 diabetes.

* Denotes time effect, *p* < .05.

^†^ Denotes group‐related difference between controls and T2D with *p*‐value listed.

^‡^ Denotes difference between 10% and 40% MVIC tasks, *p* < .05.

##### Motor unit characteristics

The mean recruitment threshold during the 40% MVIC contractions did not differ between people with T2D and controls (18.7 ± 10.9% vs. 17.4 ± 14.1% MVIC, *p* = .150), and did not change from before to after the fatiguing task (18.6 ± 12.9% vs. 18.2 ± 11.6% MVIC, *p* = .382). The mean ISI did not differ between people with T2D and controls, and the mean ISI was lower for the 40% than 10% MVIC task (*p* < .001). The mean ISI was reduced after the fatiguing task for the 40% and 10% MVIC task (*both p* < .001). This reduction in the mean ISI was not different between the groups for the 10% MVIC (time × group, *p* = .583) and 40% MVIC task (*p* = .829). The CV of ISI was greater for people with T2D than controls for both 10% and 40% MVIC tasks and the CV of ISI was greater for the 40% MVIC than the 10% MVIC task (*p* < .001). The CV of ISI increased after the fatiguing task for the 10% and 40% MVIC task (*p* < .001 and *p* = .004, respectively). This increase in CV of ISI for the 10% and 40% MVIC task was not different between the groups (*p* = .345 and *p* = .270, respectively). See Table 3.

#### 3.3.2 Knee extensor muscles

##### Torque variability

The CV of knee extensor torque was greater during the 10% MVIC task than the 40% MVIC task (*p* < .001, group and time pooled). People with T2D had a greater CV of torque compared with the controls for both contraction intensities (10% and 40% MVIC). Additionally, the CV of torque did not change after the fatiguing task for the 10% MVIC task (*p* = .156) or the 40% MVIC task (*p* = .130). See Table 3.

##### Motor unit characteristics

The mean recruitment threshold during the 40% MVIC task did not differ between people with T2D and controls (25.5 ± 9.2% vs. 24.7 ± 9.0% MVIC, *p* = .144), and did not change from before to after the fatiguing task (23.9 ± 9.7% vs. 25.3 ± 8.6% MVIC, *p* = .124). The mean ISI was lower for the 40% than 10% MVIC task (*p* < .001). For the 10% MVIC task, the mean ISI did not differ between people with T2D and controls (*p* = .723); however, for the 40% MVIC task, mean ISI was lower for people with T2D than controls (*p* < .001). The mean ISI was reduced after the fatiguing task for the 10% and 40% MVIC tasks (*both p* < .001), and this reduction in the mean ISI was not different between the groups (time × group, *p* = .173 and *p* = .730, respectively). The CV of ISI was greater for people with T2D than controls for both 10% and 40% MVIC tasks, and the CV of ISI was greater for the 40% MVIC than the 10% MVIC contraction (*p* = .001). The CV of ISI increased after the fatiguing task for the 10% and 40% MVIC task (*both p* < .001), and this increase in CV of ISI for the 10% and 40% MVIC tasks was not different between the groups (*p* = .111 and *p* = .983, respectively). See Table 3.

### 3.4 Correlation and regression models

**FIGURE 3:**
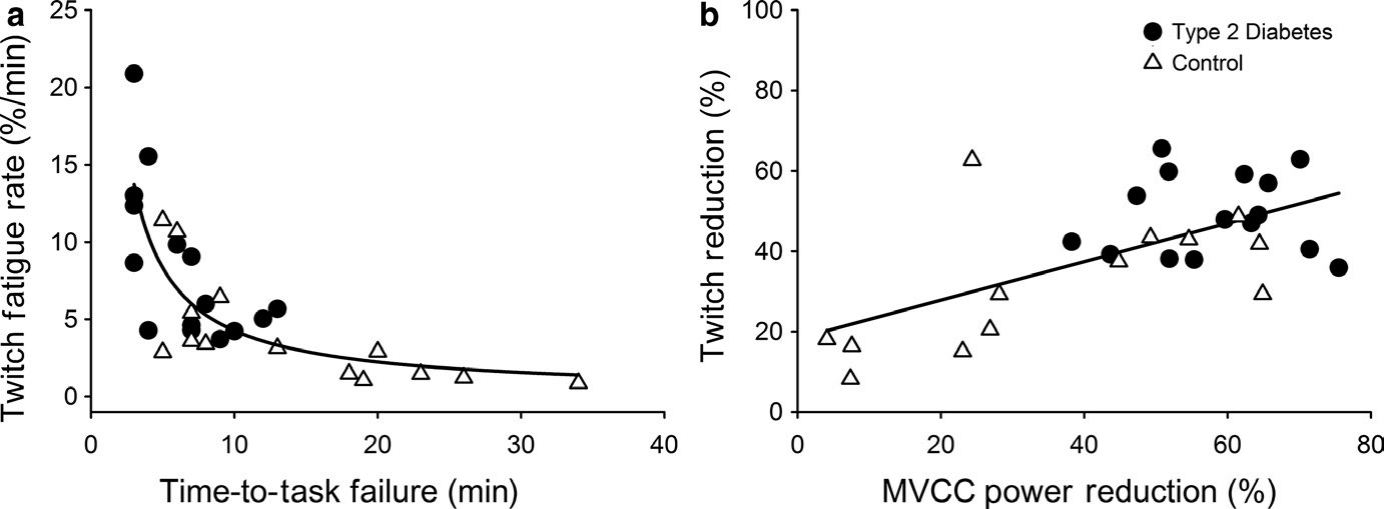
Associations with fatigability. The rate of reduction in electrically evoked twitch amplitude (%·min^‐1^) was associated with time‐to‐task failure (minutes) of the dorsiflexor muscles (a; *r* = −0.851, *r* ^2^ = 0.724, *p* < .001) and the reduction in twitch amplitude (%) was associated with the reduction in maximal voluntary concentric contraction (MVCC) power (%) of the knee extensor muscles (b; *r* = 0.655, *r* ^2^ = 0.429, *p* < .001)

For the dorsiflexor muscles, time‐to‐task failure was associated with the rates of decline in the electrically evoked twitch amplitude (‐*r* = 0.648, *r* ^2^ = 0.420, *p* < .001; Figure 3A), mean CV of ISI before the fatiguing task (*r* = 0.458, *r* ^2^ = 0.210, *p* = .014), and HbA_1c_ (‐*r* = 0.411, *r* ^2^ = 0.169, *p* = .030). Step‐wise regression modeling selected only the rate of decline in electrically evoked twitch amplitude as a predictor variable for time‐to‐task failure. CV of ISI (*p* = .145, Δ*r* = 0.051) and HbA_1c_ (*p* = .586, Δ*r* = 0.007) did not reach the required level of significance to be entered into the regression model. The optimal regression model was cubic (*r^2^* = 0.724, *p* < .001; Figure 3A).

For the knee extensor muscles, the reduction in MVCC power was associated with the reduction in electrically evoked twitch amplitude (*r* = 0.655, *r* ^2^ = 0.429, *p* < .001; Figure 3B), HbA_1c_ (*r* = 0.474, *r* ^2^ = 0.225, *p* = .006), and estimated VO_2_ (*r* = −0.394, *r* ^2^ = 0.155, *p* = .026), but not mean ISI (*p* = .261) or CV of ISI (*p* = .380). Step‐wise regression modeling selected only the reduction in electrically evoked twitch amplitude as a predictor variable for the reduction in MVCC power. HbA_1c_ (*p* = .100, Δ*r* = 0.048) and estimated VO_2_ (*p* = .679, Δ*r* = 0.003) did not reach the required level of significance to be entered into the regression model. The optimal regression model was linear (Figure 3B). Fatigability of the knee extensor muscles (reduction in MVCC power) was associated with fatigability of the dorsiflexor muscles (time‐to‐task failure; *r* = 0.379, *r* ^2^ = 0.144, *p* = .042).

### 3.5 Gender differences

Although men were stronger and more powerful than women for both groups (T2D and control), there were no gender‐related differences (main effects or interactions, *p* > .05) in any measurements of fatigability, voluntary activation, or change in contractile properties with fatigue for either muscle group (dorsiflexors or knee extensors).

For the 10% and 40% MVIC tasks (pooled), CV of torque was lower for men than women for the dorsiflexors (5.81 ± 3.86 vs. 9.73 ± 8.41%, *p* < .001) and knee extensors (2.87 ± 1.97 vs. 3.97 ± 3.75%, *p* = .002). During these isometric submaximal contractions, a larger number of motor units were identified for men than women for the dorsiflexor (2,676 vs. 1,426; *p* < .001) and knee extensor muscles (814 vs. 0; *p* < .001). However, there were no gender‐related differences (main effects or interactions) in any measurements of motor unit behavior (*p* > .05).

## 4 DISCUSSION

The novel findings of this study were that during submaximal isometric contractions with the ankle dorsiflexor and knee extensor muscles, people with T2D with no detected signs of DPN had greater variability of both motor unit discharge activity and torque (reduced steadiness) than age, body fat, and physical activity‐matched controls. People with T2D were more fatigable for both an isometric fatiguing task with the ankle dorsiflexor muscles and a dynamic fatiguing task with the knee extensor muscles compared with the controls, with no differences between males and females. The reduced steadiness and larger variability of motor unit discharges in people with T2D compared with controls were not exacerbated after the fatiguing exercises and, thus, likely did not contribute to greater fatigability of people with T2D. Rather, fatigability of both lower limb muscle groups was associated with a reduction in the potentiated twitch amplitude, providing evidence that greater fatigability of people with T2D (no signs of DPN) is due to impairments in muscle contractile properties.

### 4.1 Fatigability of lower limb muscles with T2D

In support of our hypotheses, our study demonstrated that people with T2D had greater fatigability than controls for both the dorsiflexor and knee extensor muscle groups. Our findings of greater fatigability are consistent with previous research demonstrating greater fatigability of people with diabetes (type 1 or type 2) after isometric (Allen, Kimpinski, et al., 2015; Almeida et al., 2008; Bazzucchi et al., 2015; Petrofsky et al., 2005) and dynamic (Halvatsiotis et al., 2002; Ijzerman et al., 2012; Senefeld, Harmer, et al., 2019; Senefeld, Limberg, et al., 2019; Senefeld, Magill, et al., 2018) fatiguing tasks compared to people without diabetes (prediabetes or controls). In healthy controls, the magnitude of fatigability and the relative influence of the mechanisms that contribute to fatigability are dependent on the details of the task (Enoka, 1995; Hunter, 2018), as are the differences between populations such as young versus old adults. For example, old adults are usually more fatigue resistant in lower and upper limb muscle groups for isometric tasks but this age difference is diminished during dynamic contractions (Callahan, Foulis, & Kent‐Braun, 2009; Yoon, Schlinder‐Delap, & Hunter, 2013). Furthermore, for high‐velocity contractions (similar to those performed in this current study) with the knee extensors, old adults are more fatigable than young for both males and females (Callahan & Kent‐Braun, 2011; Dalton, Power, Vandervoort, & Rice, 2012; Senefeld, Yoon, & Hunter, 2017; Sundberg, Kuplic, Hassanlouei, & Hunter, 2018). Given the task dependency of fatigability in other populations such as old adults, the data from the current study are compelling because people with T2D were twice as fatigable than controls who were matched for age, body fat, and physical activity for both an isometric and dynamic fatiguing task, as well as for two different leg muscle groups (dorsiflexors and knee extensors). There was a 56% group difference in the reduction in knee extensor power and a 51% group difference in time‐to‐task failure for the intermittent isometric task with the ankle dorsiflexor muscles. While we had previously observed greater fatigability of the knee extensor muscles in people with T2D compared to controls during the high‐velocity dynamic contractions (Senefeld, Harmer, et al., 2019; Senefeld, Limberg, et al., 2019; Senefeld, Magill, et al., 2018), the greater fatigability of people with T2D during the isometric task with the ankle dorsiflexor muscles was not known. In addition to the greater fatigability of T2D regardless of task, these findings are of particular importance because previous studies showing differences in fatigability with T2D (relative to controls) during an isometric task, the controls were not matched for gender or activity levels. Thus, these findings reveal that for fatiguing tasks with lower limb muscles, people with T2D who did not have signs of DPN were more fatigable than controls and the group‐related differences in fatigability could not be explained by activity differences or other possible confounders such as differences between the number of males and females in each group.

### 4.2 Mechanisms of fatigability

A novel finding of this study is that for both fatiguing tasks, fatigability was associated with muscle contractile properties (reduction in twitch amplitude). There were only modest changes in measures of neural activation (voluntary activation and motor unit discharge characteristics) and the indices of neural activation were not associated with fatigability. We found minimal reductions in voluntary activation (assessed with stimulation at the motor nerve) for people with T2D and controls after both fatiguing tasks. Downstream of the motor cortex, we assessed behavior of the active motoneuron pool using high‐density surface EMG isolating single motor unit behavior. The regulation of excitability of the motoneuron pool during exercise includes intrinsic adjustments of excitability properties of the motoneuron per se (e.g., persistent inward currents) as well as complex interactions of excitatory and inhibitory input from descending efferent input, spinal interneurons, and peripheral afferent feedback (Hunter, 2018). While we found that people with T2D had greater variability of motor unit firing patterns (i.e., greater CV of mean ISI) and greater variability of muscle torque during submaximal contractions before the fatiguing tasks compared with controls, this greater variability of people with T2D was *not* exacerbated after the fatiguing task for either muscle group. Thus, our data do *not* support that changes in excitability of the motor neuron pool influence fatigability of limb muscles as has been observed in people with diabetic polyneuropathy (Allen, Kimpinski, et al., 2014, 2015; Allen, Stashuk, et al., 2015). Our data do suggest, however, that the greater fatigability in people with T2D is due to mechanisms downstream of the motor neuron that disrupt contractile function within the exercising muscle groups.

The primary metrics of fatigability (reduction in power of the knee extensor muscles and time to failure of the dorsiflexor muscles) were associated with the decline in electrically evoked potentiated twitch amplitude. These findings are consistent with our previous studies showing that the reduction in the potentiated twitch explained ~ 40% of the decline in power of the knee extensor muscles over 6 min (Senefeld, Harmer, et al., 2019; Senefeld, Limberg, et al., 2019; Senefeld, Magill, et al., 2018). Although reduced muscle size and impaired muscle contractile function are commonly observed in people with T2D and diabetic polyneuropathy (Allen et al., 2013; Allen, Major, et al., 2014; Ijzerman et al., 2012), we found no reductions in muscle mass in the upper or lower leg muscles or contractile function (maximal volitional strength or electrically evoked contractile properties) of the knee extensors or dorsiflexors in people with T2D compared to controls. Thus, skeletal muscle morphology and function did not contribute to greater fatigability of people with T2D in our cohort without diabetic polyneuropathy.

The associations between fatigability and the reduction in electrically evoked twitch torque indicate that contractile mechanisms are primarily contributing to greater fatigability of people with T2D. The reduction in electrically evoked twitch torque is likely due to an additive effect of fatigue‐induced metabolic by‐product accumulation, blunted calcium (Ca^2+^) sensitivity, and blunted Ca^2+^ transients (Debold, Fitts, Sundberg, & Nosek, 2016); however, the precise role of metabolic by‐products in human fatigability remains a topic of debate even in healthy muscle (Fitts, 2016; Westerblad, 2016). Potential mechanisms contributing to greater disruption of the skeletal muscle contractile function in people with T2D could be due to a number of possible reasons, including lower proportions of fatigue‐resistant type I muscle fibers (Stuart et al., 2013), reduced skeletal muscle blood flow during exercise (Senefeld, Limberg, et al., 2019), smaller and less efficient mitochondria (Kelley et al., 2002), or reduced concentrations of substrates for metabolic processes to sustain muscle performance (e.g., ATP and PCr (Ripley et al., 2018; Ritov et al., 2010)). Although the precise mechanisms are unknown, it is important to recognize that many of these potential mechanisms could be rectified with regular exercise training (Laughlin, 2016; Meex et al., 2010), and the contribution of these mechanisms to greater fatigability of people with T2D warrant future investigations.

### 4.3 Steadiness and motor unit behavior

A novel finding of this study was that people with T2D who did not have signs of DPN and were matched with controls for age, body fat, and physical activity, were less steady during low‐ and moderate‐intensity isometric contractions. Thus, the greater variability in the torque signal was not attributed to differences in activity levels but associated with the disease of T2D. The group‐related difference in variability of torque was similar between the 10% and 40% MVIC tasks for the dorsiflexor (10% vs. 15%) and knee extensor muscles (25% vs. 20%). Consistent with our hypotheses, the magnitude of torque variability and the group‐related differences in torque variability were both greater for the dorsiflexor than the knee extensor muscles. Thus, the greater variability of torque in people with T2D compared with controls is exacerbated in the distal muscle group (dorsiflexors) which suggests that greater variability of torque during submaximal muscle contractions of the lower limb may be a sign of subclinical changes in the motor neurons. The steadiness in torque during submaximal contractions is primarily modulated by common synaptic input to the lower motor neurons (Negro, Holobar, & Farina, 2009; Negro, Yavuz, & Farina, 2016b), thus, these data suggest greater variability of low‐frequency common synaptic oscillations in neural drive among people with T2D.

Additionally, our data support that motor unit characteristics are more variable in people with T2D in two lower limb muscle groups (dorsiflexors and knee extensors), as has been observed in the knee extensors previously (Watanabe et al., 2013). Variability of motor unit discharge (CV of ISI) was greater for people with T2D compared to controls; however, this group‐related difference was not different between contraction intensities (10% vs. 40% MVIC) or muscles (vastus lateralis vs. tibialis anterior). Thus, irrespective of contraction intensity or muscle group, people with T2D had greater variability of single motor unit discharge rate, which may indicate early, subclinical signs of change in the properties of the motor unit.

## 5 CONCLUSIONS

The present study provides novel evidence that people with T2D without clinically detected DPN have greater variability in both torque and motor unit characteristics compared with controls matched for age, body fat, and physical activity. The greater variability of torque of people with T2D was exacerbated in the more distal muscle group (dorsiflexor vs. knee extensor muscles), which suggests that the increased variability may be due, at least in part, to subclinical manifestations of DPN. People with T2D also had greater fatigability of the dorsiflexor and knee extensor muscles compared to controls, however, the greater fatigability was not associated with variability of torque or motor unit characteristics. Rather, fatigability of both lower limb muscle groups was associated with a reduction in the potentiated twitch amplitude. Thus, we conclude that the increased fatigability of people with T2D of lower limb muscles during both dynamic and isometric fatiguing tasks is associated with greater impairments in contractile function of the exercising muscles.

## CONFLICT OF INTEREST

No conflicts of interest, financial or otherwise, are declared by the authors.

## AUTHOR CONTRIBUTIONS

J.W.S., K.G.K., and S.K.H. conceived and designed research; J.W.S., K.S.R., and S.E.D. performed experiments; J.W.S., K.G.K., K.S.R., and F.N. analyzed data; J.W.S., K.G.K., F.N., and S.K.H. interpreted results of experiments; J.W.S. and K.S.R. drafted manuscript; J.W.S., K.G.K., F.N., and S.K.H. edited and revised manuscript; J.W.S., K.G.K., K.S.R., S.E.D., F.N., and S.K.H. approved final version of manuscript; J.W.S. prepared figures.
